# Sunflower (*Helianthus annuus* L.) Seed Hull Waste: Composition, Antioxidant Activity, and Filler Performance in Pectin-Based Film Composites

**DOI:** 10.3389/fnut.2021.777214

**Published:** 2021-12-15

**Authors:** Maria D. De'Nobili, Dana C. Bernhardt, Maria F. Basanta, Ana M. Rojas

**Affiliations:** ^1^Departamento de Industrias-Instituto de Tecnología de Alimentos y Procesos Químicos (ITAPROQ), Facultad de Ciencias Exactas y Naturales, Universidad de Buenos Aires, Buenos Aires, Argentina; ^2^Consejo Nacional de Investigaciones Científicas y Técnicas (CONICET), Buenos Aires, Argentina; ^3^Instituto de Tecnología-INTEC, Universidad Argentina de la Empresa (UADE), Buenos Aires, Argentina

**Keywords:** sunflower seed hull, chemical composition, antioxidant capacity, seed hull film composites, water vapor permeability, tensile strength

## Abstract

*Helianthus annuus* L. seed hull is an abundant waste of the edible oil industry. To envisage potential applications of this waste, here, we aimed to analyze the chemical composition of milled sunflower hulls (SP), constituted mainly by 210 μm (51.4%) and 420 μm (27.6%) average mesh particle sizes. SP contained almost 30% of cellulose, 26.4% of lignin, 38.5% of neutral sugars, mainly hemicelluloses, and only 1.3% of proteins. The important lignin content and low pectin content (4.0% of uronic acids) present in SP were both ascribed to its low hydrophilic behavior and hydration capacity. Phenolic compounds were mostly proanthocyanidins (168 mg/100 g SP), with lower amounts of extractable (31.4 mg/100 g SP) phenolics (*O*-caffeoylquinic acid), all of them associated with the DPPH radical scavenging capacity (95 mg ascorbic acid equiv./100 g) and ferric reducing power (FRAP: 152 mg ascorbic acid equiv./100 g) shown by SP. Esterified ferulic acid (52.9 mg/100 g SP) was also found, mostly as monomers and trimers. SP of 53 μm particle size was then assayed as a filler (0, 5, 8, and 12% concentrations) in calcium low methoxyl pectin-based films, which showed antioxidant capacity (DPPH and FRAP assays) in an SP-concentration-dependent manner. SP showed homogeneous dispersion in composite films equilibrated at 57.7% relative humidity. Water content decreased while film thickness increased with SP concentration. When loaded at a 12% level, the presence of 53-μm SP decreased the water vapor permeability and increased the normal stress at film fracture. Sunflower hulls can then be applied to the development of active materials like 12% SP film, which can be proposed as a food slice antioxidant separator to be investigated in a future work.

## Introduction

Sunflower (*Helianthus annuus* L.), which belongs to the family Asteraceae, is an annual short season herbaceous crop with more than 70 species known worldwide. It is characterized by a typical large circular yellow flower head inflorescence (bearing achenes developing into mature seeds) facing directly the Sun and is original from native temperate climates (20–25°C temperature range) of North America (1, 2).

Sunflower is one of the world's leading oilseed crops (3). In 2020/2021, the world's production of sunflower oilseed was 49.60 million metric tons whereas that of sunflower oil was 19.30 million metric tons. In Argentina, the production of sunflower oilseed in the same period was 1,225 thousand metric tons (4). This country has regions with excellent conditions for the growth of sunflowers. Sunflower oil, together with palm, soybean, and rapeseed oil, accounts for around 87% of the world's edible oil production (2). The sunflower seed hull is an abundant waste of the edible oil industry. In Argentina, the average sunflower seed production is about 3.5 million tons per year. However, about 50% of the seed weight generates a solid lignocellulosic residue, which is underused and stored in the open air, used for landfill, or burnt. Environmental concerns related to this waste and other agroindustrial wastes have stimulated the development of improved technologies for waste treatment. These technologies include the thermal conversion of biomass residues into high-value products such as energy, biochar, biochemical feedstock, and liquid transportation fuels (5). However, basic valorization strategies such as the production of animal feed (6), burning for energy, composting, and recycling use <50% of the wastes. In contrast, advanced valorization strategies, especially those based on green chemical technologies, can diversify the generation of multiple products by extraction of valuable components or chemicals (7, 8). In this context, the sunflower hulls remaining after oil extraction can constitute an alternative carbon source of useful biopolymers and natural antioxidants for materials, dietary fibers, additives, and/or ingredients for food preservation. Toxicity analysis of sunflower hull based on the brine shrimp lethality assay has shown chronic toxicity as low as 1 g/mL (6). This is an extremely important quality since this waste can then be applied to the development of edible materials such as active films. An edible film is any self-supporting material with a thickness below 0.3 mm and formed by biopolymers and different additives dispersed in aqueous media. Active edible films can be produced by loading food preservatives such as antioxidants, UV-light protectors, antimicrobial agents, and pigments (9). Compartmentalization into an edible film can not only stabilize food preservatives but also contribute to overcome negative interactions between these compounds as well as with other food components. A localized activity of the food preservative loaded in films can be also the way to achieve higher efficiency and to enhance availability (10).

To establish the potential of sunflower hulls to constitute an alternative carbon source, the aims of the present study were: (i) to analyze their chemical composition and (ii) to assay them as fillers (0, 5, 8, and 12% concentrations) to decrease the water vapor permeability (WVP) and improve the surface characteristics and mechanical strength of calcium-crosslinked low methoxyl pectin-based films, and to evaluate the sunflower hull ability to provide these films with antioxidant activity for future applications in food preservation.

## Materials and Methods

### Chemicals

Deionized water (MilliQ, Millipore, USA) was used. Food grade pectin with a low degree of methyl esterification (GENU™ pectin type LM-12 CG) was a gift from CP Kelco (Denmark). Potassium sorbate, bovine serum albumin standard, D-galacturonic acid, 2,2-diphenyl-1-picrylhydrazyl (DPPH), 2,4,6-tripyridyl-s-triazine (TPTZ), and FeCl_3_.6H_2_O were obtained from Sigma–Aldrich (St. Louis, USA). The other chemicals were of analytical quality from Merck (Argentina).

### Sunflower Seed Hull Powder (SP)

Sunflower seed hulls (*Helianthus annuus* L.) discarded after oil extraction were donated by Cargill Argentina S.A.C.I. Hulls were dried at 65°C for 4 h under high air convection and then crushed in a cutting mill (SM 300, Retsch, Germany) provided with a bottom sieve of 500-μm mesh size. For particle size characterization, a given weight of the milled hulls was sieved through a vibratory sieve shaker (Retsch, Germany) provided with 840, 420, 210, 105, 53, and 25 μm ASTM mesh sizes. The fraction recovered between the two closest sieves was weighed to calculate the yield (% w/w). Finally, the whole hull powder was packed under vacuum into hermetically sealed Cryovac™ pouches. On the other hand, the 53-μm hull powder fraction was afterward separated and used for film development.

### Chemical Analyses

Cellulose, lignin, and non-cellulosic polysaccharides were separated with sulfuric acid aqueous solutions (1 M or 72% w/w) based on the method of Martín-Cabrejas et al. (11), as explained in Basanta et al. (12) and Aramburu et al. (13). Cellulose and lignin were separately quantified by weighing the respective insoluble residues obtained after extraction of SP (0.3000 g) with either (a) 1 M-sulfuric acid (25 mL) for 2.5 h at 100°C in a water bath or (b) 72% w/w sulfuric acid (2.08 mL; 3 h; room temperature). The treatment (b) was completed by the addition of enough deionized water to achieve 1 M sulfuric acid (total volume made 25 mL), and maintained at 100°C for 2.5 h in a water bath with periodic mixing, maintaining constant the total volume by addition of boiling deionized water. In treatment (a), the non-cellulosic polysaccharides (pectins and hemicelluloses) are dissolved, while cellulose and lignin remained in the residue. Instead, in treatment (b), the non-cellulosic polysaccharides and cellulose are dissolved, but not lignin (11–13). The respective residues obtained after (a) or (b) treatment were carefully and repeatedly washed until a pH of 5.8 was reached by dispersion in deionized water, followed by centrifugation, being each supernatant discarded. The washed residue finally obtained was freeze-dried and then weighed, obtaining the lignin content after treatment (b). Instead, the cellulose content was calculated as the arithmetical difference between the weights of the residues obtained through treatment (a) and treatment (b). In the supernatant obtained after treatment (b) (72% w/w and 1 M sulfuric acids), the UA content of pectins was determined through the colorimetric method of Filisetti-Cozzi and Carpita (14), and the total polysaccharide content (non-cellulosic polysaccharides + cellulose) through the phenol-sulfuric acid spectrophotometric method of DuBois et al. (15), in both cases using D-galacturonic acid as standard. The content of neutral sugars (NS) was calculated as the arithmetical difference between the total non-cellulosic polysaccharides and the UA contents.

The protein content was determined through the method of Lowry et al. (16) as reported in Basanta et al. (12), using bovine serum albumin as standard.

### Phenolic Compounds in SP

#### Extraction

Approximately 2.5 g of SP was sonicated for 15 min with 20.0 mL of extractive solution [acetone/water/acetic acid; 70:29.5:0.5 (v/v/v)] (17). The homogenates were then centrifuged at 1,765 × *g* for 10 min (JP Selecta Centronic, Barcelona, Spain). The acetone was evaporated at 35.0°C under reduced pressure. The aqueous residue was filtered through an activated Sep-Pack C-18 solid phase extraction cartridge (Waters, Milford, MA, USA), washed with water, and the retained phenolic compounds were then eluted with 1,000 μl of methanol, filtered through a 0.45-μm nylon filter and directly analyzed by high performance liquid chromatography (HPLC).

### Analysis, Quantification, and Identification of Extractable Phenolic Compounds

The phenolic compounds were identified through HPLC-PAD-ESI-microTOF/MS as explained in Aramburu et al. (13) with an Agilent 1200 HPLC system (Agilent Technologies, Wilmington, USA) provided with a binary pump (model G1312B), an autosampler (model G1367D), a degasser (model G1379B), and a PAD (model G1315C). Spectral data from all peaks were accumulated in the 200–800 nm range. The HPLC equipment was coupled with a high-resolution mass spectrometer Bruker microTOF-QII (Bruker Daltonics, Billerica, MA, USA) with an electrospray ionization source (ESI).

The polyphenols identified were quantified with a Waters 1525 HPLC system (Milford, MA, USA) equipped with a binary pump and photodiode array detector (PAD). A C18 column (250 × 4.0 mm, 5 μm particle size; Luna, Phenomenex, USA) was used. The mobile phase was composed of water (1% formic acid) (A) and methanol (B), and the flow rate was 0.8 mL/min and 20 μL injection volume. A linear gradient starting with 10% of B until reaching 30% at 20 min was used. Spectral data from all peaks were accumulated in the 200–400 nm range. The content of each phenolic compound in the SP was expressed as mg/100 g of SP.

### Analysis and Quantification of Proanthocyanidins

Flavan-3-ols were determined as reported in Aramburu et al. (13), using the phloroglucinolysis reagent constituted by a 0.1 N HCl-methanolic solution, containing 50 g/L of phloroglucinol and 10 g/L of L-(+)-ascorbic acid. Approximately 50 mg of SP was treated with the phloroglucinolysis reagent (800 μl) for 20 min in a water bath at 50°C. The reaction was stopped by placing the vials in an ice bath and diluting the reaction medium with 1 mL of a 40 mM sodium acetate solution.

The phloroglucinol adducts were then analyzed by HPLC–PAD–MS/MS using a C18 column (250 × 4.0 mm, 5 μm particle size; Luna, Phenomenex, USA). The method used a binary gradient with 2.5% v/v aqueous acetic acid (mobile phase A) and acetonitrile (mobile phase B), at a flow rate of 1 mL/min. The linear gradient started with 3% B, at 5 min 5% B, at 15 min 16% B, at 45 min 50% B, and at 52 min 3% B, and was maintained in isocratic condition up to 57 min. Flavan-3-ol cleavage products were estimated using their response factors relative to catechin, which was used as the quantitative standard. The apparent degree of polymerization (DPn) was calculated as the sum of all subunits (flavan-3-ol monomers and phloroglucinol adducts, mg) divided by the sum of all flavan-3-ol monomers (mg).

### Analysis of Esterified Phenolic Compounds

Covalently linked phenolic compounds were extracted through alkaline hydrolysis (1 g powder: 15 mL 2 M NaOH) by stirring under vacuum in darkness, at 4°C for 2 h, according to Vaidyanathan and Bunzel (18). Afterward, the pH was adjusted to 1.5 by the addition of 6 M HCl. Three successive extractions with 25 mL of ethyl acetate were performed, and then the supernatants were combined, and the solvent was evaporated at low pressure (Büchi rotavap, Germany). The residue was dissolved by the addition of 1,000 μl of methanol, filtrated through a 0.45-μm membrane, and analyzed by reversed-phase HPLC-PAD and HPLC-ESI-MS/MS, as explained earlier.

### Hydration Properties of SP

The swelling capacity (SC), the water holding capacity (WHC), and the water retention capacity (WRC) of the SP were separately determined after hydration for 18 h at 25°C in an excess measured volume of deionized water, as described in Idrovo Encalada et al. (19).

### Film Preparation

The 53-μm average mesh particle size fraction of SP was afterward separated and used for film development. According to the 53-μm SP concentration considered (0.0, 5.0, 8.0, or 12.0 g per 100 g of low methoxyl pectin) for film making, fractions of 0, 0.2625, 0.4200, and 0.6300 g of 53-μm SP were, respectively, dispersed in 20.00 mL of deionized water, mixed through a vortex for complete hydration, and left for 24 h. Separately, 8.00 g of the commercial GENU™ pectin containing 5.25 g of low methoxyl pectin was dispersed in ≈260.00 g of deionized water held in a glass beaker, under continuous controlled high-speed shear (1,400 rpm-constant) stirring performed with a vertical stirrer (LH, Velp Scientifica, Italy) for homogeneous hydration of the pectin powder. Afterward, heating (5°C/min) up to 90°C was achieved under stirring. Potassium sorbate (0.0900 g) as an antimicrobial agent and glycerol (5.00 g) as a plasticizer, previously dissolved and mixed in ≈15 ml of deionized water, were then added. The dispersion (20.00 mL) of a given 53-μm SP concentration was then added to the film-making solution while stirring, and, finally, 0.5000 g of CaCl_2_.2H_2_O pre-dissolved in ≈5 mL of deionized water was added, maintaining the system at 85°C with stirring. The total weight of the film-making solution was then made 300.00 g by the addition of enough deionized water and then homogenized (13). This hot solution was placed under vacuum for 20 s to remove air bubbles and poured onto horizontally leveled and numbered polystyrene plates (55 mm in diameter) at a constant weight. After cooling at a low temperature (8°C) for gelling, the solution was dried (60°C, 2.5 h) under forced convection. Films were then peeled off and stored hanged under vacuum (25.0°C) in chambers containing a NaBr saturated solution (water activity, $a_{\text{W}}^{∙}$ = 0.577), to maintain a constant equilibrium relative humidity (ERH) of 57.7% for film equilibration (Equation 1) (14).

Three batches of films for each 53-μm SP concentration studied (0.0, 5.0, 8.0, and 12.0 %) were produced as explained, and films obtained were numbered.

Film *a*_W_ was daily measured in triplicate at 25.0°C through a Decagon AquaLab (Series 3 Water activity meter, USA), to determine the time at which ERH was attained (Equation 1).


(1) $$\begin{array}{l} a_{W}{=\;}^{ERH}{/}_{100} \end{array}$$

### SP and Film Characterization

The following assays were performed on at least one film sample from each batch after ERH (57.7%) was reached (Equation 1).

#### Antioxidant Capacity

Antioxidant capacity was determined through the radical scavenging activity of the SP or cut film samples by using the DPPH assay (20), and also through the ferric reducing antioxidant power (FRAP) assay reported by Benzie and Strain (21) and Pulido et al. (22). Samples were extracted with methanol and results were expressed on L-(+)-ascorbic acid (AA) as the standard in both methods, whose calibration curves were developed with the standard dissolved in methanol.

#### Color

SP was placed into a 20-mm diameter transparent and colorless cell. In the case of films, the exposed area was sufficiently large in relation to the illuminated area to avoid any edge effect (19). A Minolta colorimeter (CM-600D, Tokyo, Japan) provided with a 1.5-cm diameter aperture was used at six different points across the sample surface. Hunter Lab color parameters like lightness [*L* = 0% (black) and *L* = 100% (white or maximum)], *a* [−*a* (greenness) to +*a* (redness)], and *b* [*-b* (blueness) to +*b* (yellowness)] were determined using D65 standard illuminant, and 2° standard observer. The average and standard deviation for triplicates of SP or films are reported.

#### The Water Content of Films

The water content of film samples cut in 1-mm pieces was determined by drying under vacuum at 70°C up to constant weight (≈34 d). It was determined in triplicate, and then calculated and expressed per 100 g of dry mass.

#### Glass Transition Temperature (T_g_)

Modulated differential scanning calorimetry (MDSC, TA Instruments, USA) was used to determine the *T*_g_ (midpoint temperature) as the first derivative of the heating ramp recorded from the second scan performed on a 57.7% RH equilibrated film sample or 57.7% RH equilibrated 53-μm SP powder sample (10–15 mg) loaded into a hermetically sealed alumina pan, as explained in De'Nobili et al. (23).

#### Water Vapor Permeability of Films

The WVP of films was determined and calculated according to the correction of Gennadios et al. (24) for hydrophilic films. Each film sample was placed between the anhydrous calcium chloride in the cup container and an environment with 70% RH, at 25°C (Ibertest chamber, Spain).

#### Tensile Assays of Films

The film sample strips (25.0 mm × 6.0 mm) equilibrated at 57.7% RH with parallel sides were cut with a cork borer and each probe was subjected to a uniaxial tensile assay until film rupture (5 mm/min-constant crosshead speed), using an Instron Testing Machine (model 3345, Norwood, MA, USA) provided with a 100 N load cell and pneumatic clamps with parallel faces coated with rubber. Film thickness was measured with a digital micrometer (Mitutoyo, Kawasaki, Japan) to the nearest 0.001 mm at three different locations of the specimen before performing each tensile assay.

Force (N)–elongation (m) curves were recorded and the normal stress (σ_*break*_) and strain (ε_*break*_) at film break or fracture were, respectively, calculated as:


(2) $$\begin{array}{l} \sigma_{break}=F_{break}/A_{O} \end{array}$$

(3) $$\begin{array}{l} \varepsilon_{break}=\left(L_{break}-L_{O}\right)/L_{O} \end{array}$$

wherein *A*_O_ is the initial cross-sectional area of the film sample; *L*_O_ is the initial gap between the clamps that maintained fixed the film probe with a pre-load of 0.1 N; and *F*_*break*_ and *L*_*break*_ are the film force and the film length, respectively, reached film fracture.

At least 10 film strips were measured for each SP concentration.

#### The Contact Angle of Films

The contact angle was tested through the sessile drop (0.0040 ml) deposited at three representative areas of each horizontally leveled film surface (three film samples per system studied) using a contact angle goniometer (NRL Contact Angle Goniometer, model 100-00, Rame-Hart, USA), as explained in Idrovo Encalada et al. (19).

#### Scanning Electron Microscopy of Films

For morphological characterization, films were cut in rectangular pieces of about 9 × 40 mm, immersed in liquid nitrogen, and then mounted vertically on the stationary support for observation of the surface morphology. Samples were then gold-coated in a chamber under a high vacuum and afterward placed into the chamber of an FEI Quanta 250 FEG scanning electron microscope (Thermo Fischer Scientific, USA). Images were acquired under a high vacuum, at an accelerating voltage of 3 kV, and using an ETD detector for secondary electrons.

### Statistical Analysis

Results are informed as to the average and SD for *n* replicates of each sample. Statistical analyses were performed through ANOVA (level of significance, α: 0.05) followed by multiple comparisons evaluated through the least significant difference test, and *p* < 0.05 represented a significant difference (Statgraphic Plus, Manugistic Inc., USA).

## Results and Discussion

### Sunflower Seed Hull Powder (SP)

#### Characteristics

**Figure 1:**
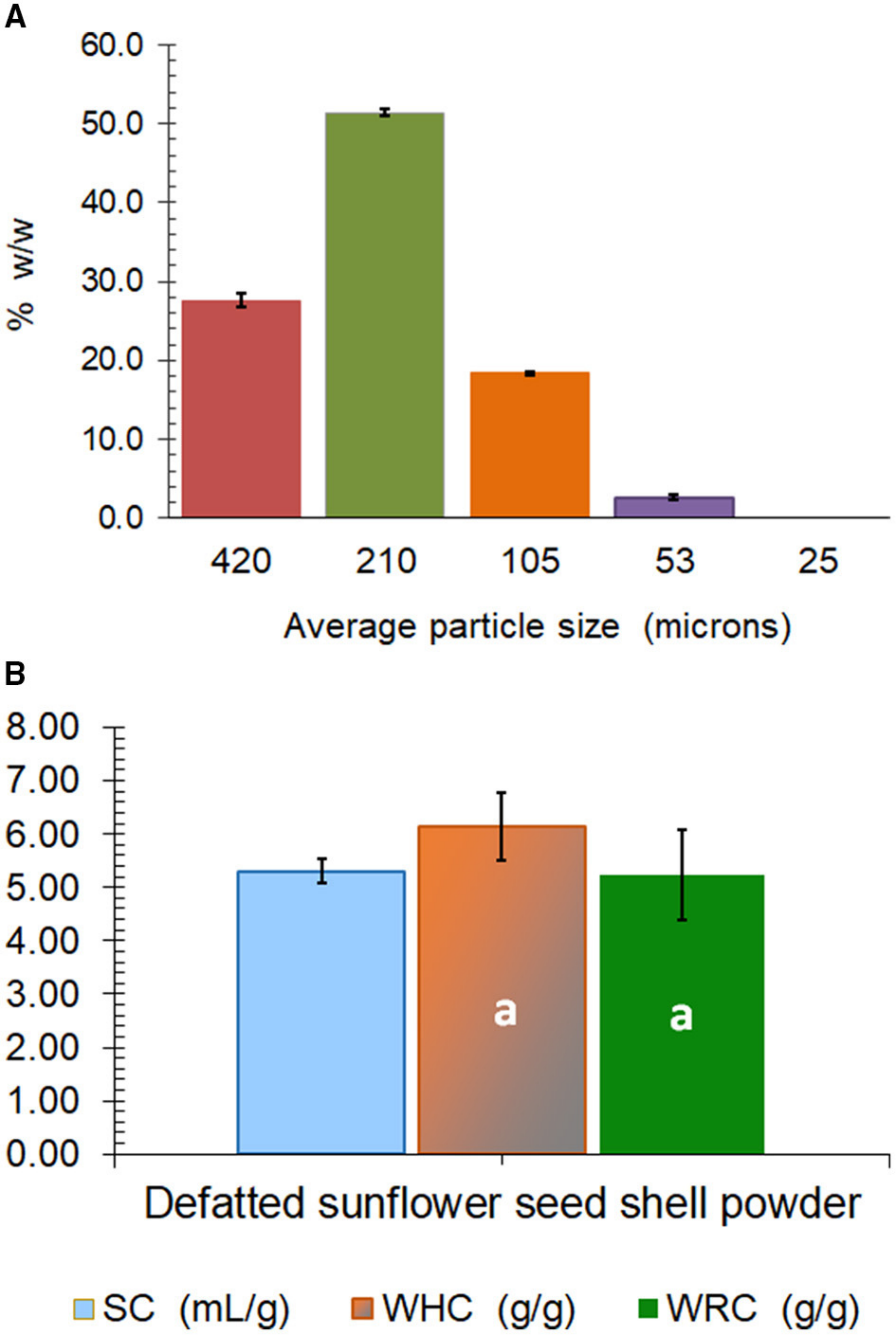
Mesh average particle size composition **(A)**, and hydration properties (SC, swelling capacity; WHC, water holding capacity; WRC, water retention capacity) **(B)** of sunflower seed hull powder (SP). The same lowercase letter over the bars indicates non-significant differences (*p* > 0.05) between WHC and WRC. Error bars correspond to the standard deviation (*n* ≥ 3).

The powder obtained after milling of sunflower seed hulls, called SP, had a water activity of 0.575 at 25°C (Table 1) and was constituted by 51.4% w/w of particles with 210 μm, 27.6% of particles with 420 μm, 18.3% of particles with 105 μm, and 2.6% of the particles with 53 μm of average mesh size (Figure 1A). The Hunter Lab color parameters of the powder showed low lightness (*L* = 35.4%) together with *a* and *b* values of +1.90 and +6.81, respectively, which corresponded visually to a dark gray-slightly reddish color (Table 1). Results also showed that SP was chemically constituted by 1.3% w/w of proteins, almost 30% of cellulose and 26.4% of lignin, 4.0% of uronic acids of pectins, and 38.5% of neutral sugars, which can comprise mainly the monosaccharides that constitute the hemicelluloses, but also low proportions corresponding to the rhamnogalacturonans of pectins (Table 1). The molar ratio of neutral sugars to uronic acids is lower than 1.5 for pectins (12).

**Table 1:** Water activity^a^, Hunter Lab color parameters^a^, chemical composition^a^, and antioxidant capacity^a^^,^^b^ obtained from sunflower seed hull powder (SP).

	**SP**
Water activity (25.0°C)	0.575 ± 0.002
*L* (%)	35.4 ± 0.2
*a*	+1.90 ± 0.01
*b*	+6.81 ± 0.03
Proteins (% w/w)	1.3 ± 0.1
Uronic acids (% w/w)	4.0 ± 0.5
Neutral sugars (% w/w)	38.5 ± 0.1
Cellulose (% w/w)	29.8 ± 0.7
Lignin (% w/w)	26.4 ± 0.5
DPPH (mg AA/100 g powder)	95 ± 2
FRAP (mg AA/100 g powder)	152 ± 5

a *Mean and standard deviation for n = 3 are reported*.

b *Results of the DPPH and FRAP assays are expressed as L-(+)-ascorbic acid (AA)*.

Regarding the hydration properties, an SC of 5.3 cm^3^ of swelled SP per gram of defatted SP was determined, while the WHC (6.1 g water/g powder) was not significantly different from the WRC (5.2 g water/g powder) (Figure 1B). These values of hydration properties are low, as usually observed for fibers obtained from cereals. The high content of the hydrophobic lignin together with the very low content of pectins (4% of uronic acids; Table 1) can be responsible for the low hydrophilic behavior of the SP (Figure 1B). Wheat bran, which is an essential byproduct of wheat milling that contains about 45–53% of dietary fiber, shows values of SC, WHC, and WRC that range between 3.50 and 7.67 cm^3^/g, 3.39 and 6.49 g water/g, and 2.17 and 5.76 g water/g, respectively (25). Similarly, microfibril wheat bran obtained through the wet smashing of wheat bran using a colloidal mill for higher palatability has a mean particle size of 35 μm and a WHC of 5.1 g/g (26).

#### Phenolic Compounds of SP

**Figure 2:**
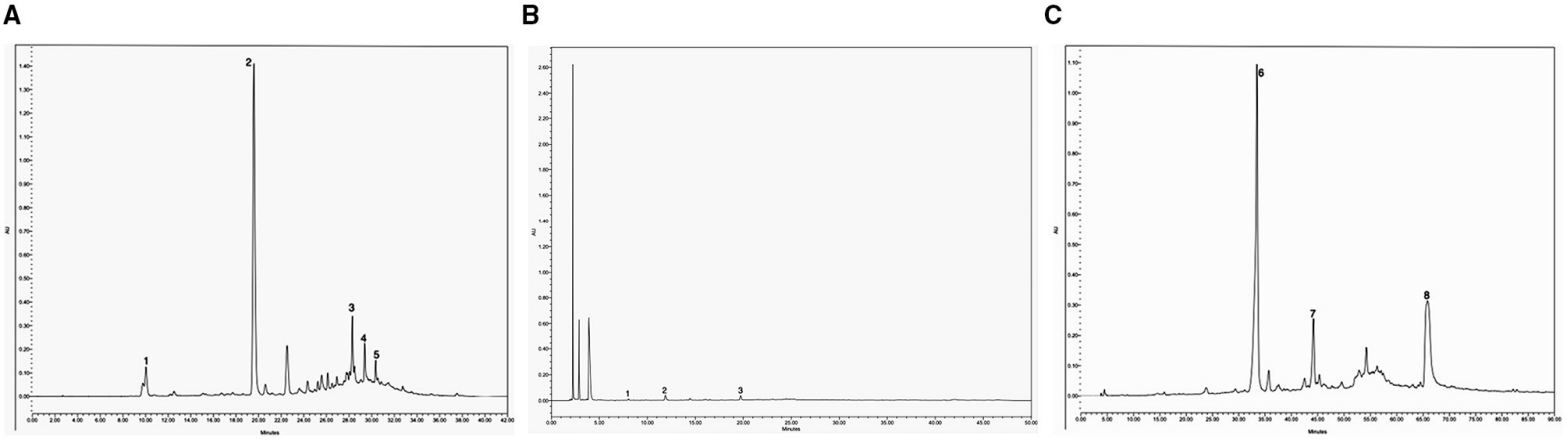
HPLC chromatograms were obtained from SP at 320 nm of wavelength for extractable phenolic compounds. Peaks: 1, *O*-caffeoylquinic acid; 2, *O*-caffeoylquinic acid isomer; 3, caffeoyl-dimethoxycinnamoylquinic acid; 4, caffeoyl-dimethoxycinnamoylquinic acid isomer; 5, dicaffeoylquinic acid **(A)**. HPLC-DAD chromatogram (280 nm) of proanthocyanidin phloroglucinolysis breakdown products obtained from SP: 1, epicatechin extension units; 2, catechin terminal units; 3, epicatechin terminal units **(B)**. HPLC chromatograms were obtained from SP at 320 nm of wavelength for esterified phenolic compounds. Peaks: 6, ferulic acid monomer; 7, ferulic acid dimer, 8, ferulic acid trimer **(C)**.

The phenolic compounds of the SP were first analyzed in their extractable fraction obtained with acetone/water/acetic acid as described above, which showed a total content of 31.4 mg/100 g SP (Table 2). These compounds absorbed in the UV region of the spectrum (Figure 2A), being hydroxycinnamic acids of two groups: monoacyl quinic (peaks 1 and 2; Table 2) and diacyl quinic (peaks 3–5; Table 2) acids. In the former group, two isomers of the *O*-caffeoylquinic acid were identified through HPLC-MS/MS, one of which (peak 2: retention time = 19.5 min; λ_max_ = 326 nm; [M–H]^−^ at m/z 353, 191, 179) was the main component of the extractable phenolics, with a content of 23.7 mg/100 g SP (Table 2). In contrast, very low contents of two isomers of the caffeoyl-dimethoxycinnamoylquinic acid were identified through HPLC-MS/MS with somewhat different retention times (peaks 3 and 4: 28.2 and 29.3 min, respectively), and also of dicaffeoylquinic acid (peak 2) (Figure 2A), with a retention time of 30.3 min, a maximum absorbance (λ_max_) at 321 nm, and [M–H]^−^ at m/z values of 515, 353, and 179 (Table 2). The analysis performed by HPLC-ESI-microTOF/MS and HPLC-PAD did not allow verifying which peak corresponded to each isomer. One of them could be 3,5-dicaffeoylquinic acid, while the other could be 4,5-dicaffeoylquinic acid, as reported by Martini et al. (27). Karamać et al. (28) analyzed the phenolic composition of 80%-methanolic extracts obtained from non-oil type dehulled and defatted sunflower seeds or sunflower kernels and found caffeoyl-dimethoxycinnamoylquinic acid and dicaffeoylquinic acid, as well as 3-*O*-caffeoylquinic acid, 4-*O*-caffeoylquinic acid, and 5-*O*-caffeoylquinic acid, among others. Similarly, Weisz et al. (29) extracted phenolics with 60% methanol from defatted hulls of sunflower seeds and determined the presence of 2.9, 2.1, and 26.6 mg of 3-*O*-caffeoylquinic, 4-*O*-caffeoylquinic, and 5-*O*-caffeoylquinic acids, respectively, per 100 g of hulls, as well as of 1.2, 4.8, and 2.6 mg of dicaffeoylquinic acids, including 3,4-, 3,5-, and 4,5-di-*O*-caffeoylquinic acids, per 100 g of hulls. These authors also found low amounts of other phenolics such as caffeic and ferulic acids (0.8 mg/100 g) as well as of 5-*O*-*p*-coumaroylquinic and 5-*O*-feruloylquinic acid (2.6 mg/100 g).

**Table 2:** HPLC-DAD and HPLC-ESI-MS results of the extractable and esterified phenolic compounds found in the sunflower seed hull powder (SP).

**Phenolic compound**	**Peak number**	**mg/100 g**	**Retention time (min)**	**HPLC-UV-DAD λ_max_ (nm)**	**HPLC-ESI-MS/MS [M-H]^−^ (*m*/*z*)**
Extractable phenolic compounds					
**Monoacyl quinic acids**					
*O-*Caffeoylquinic acid	**1**	3.0 ± 0.8	10	327	353
*O-*Caffeoylquinic acid isomer	**2**	23.7 ± 0.8	19.5	326	353, 191, 179
**Diacyl quinic acids**					
Caffeoyl-dimethoxycinnamoylquinic acid	**3**	2.2 ± 0.3	28.2	329	543, 381
Caffeoyl-dimethoxycinnamoylquinic acid isomer	**4**	1.6 ± 0.4	29.3	329	543, 381
Dicaffeoylquinic acid	**5**	0.9 ± 0.7	30.3	321	515, 353, 179
Total content of extractable phenolics		31.4			
Esterified phenolic compounds					
Ferulic acid monomers	**6**	34.0 ± 0.7	33	244/323	193
Ferulic acid dimers	**7**	3.5 ± 0.5	44	245/322	385
Ferulic acid trimers	**8**	15.4 ± 0.8	66	327	563
Total content of non-extractable (esterified) phenolics		52.9			

In a second analysis, in the present study, we investigated the presence of proanthocyanidins (condensed tannins with flavan-3-ol subunits) following acid catalysis in the presence of an excess of phloroglucinol coupled with the reversed-phase HPLC-MS-MS, as previously reported (30). We found that proanthocyanidins were the main components of the extractable phenolic compounds of SP, with a total content of 168 mg/100 g SP (Table 3). Their degree of polymerization (DPn) was 1.25. As summarized in Table 3, the proanthocyanidins of SP were constituted by flavan-3-ols such as (–)-epicatechin as extension units (26.8%) (peak 1), and (+)-catechin (35.1%) (peak 2) and (–)-epicatechin (38%) (peak 3) terminal units. The chromatogram is shown in Figure 2B, which was similar to that obtained in the analysis of Japanese plum (*Prunus salicina*) microparticles (12). To the best of our knowledge, this is the first study to determine the presence of proanthocyanidins as the main components in sunflower hulls. This is an important finding because proanthocyanidins can relieve nutrition-induced decreases in luminal small IgAs, the primary immunological barrier against pathogens (31). In sorghum bran extracts rich in proanthocyanidins, Zhu et al. (32) identified oligomers, including monomers, dimers, trimers, tetramers, pentamers, and hexamers with antioxidant activity and the ability to inhibit the proliferation and migration of HepG2 cancer cells through activation of the AMPKα pathway and inhibition of the mitogen-activated protein kinase pathway. Instead, only dimers, and occasionally trimers, have been found in the systemic circulation but at very low levels after consumption of vegetable products (33). In our work, a DPn of 1.25 means that the proanthocyanidins found in SP (Table 3) are dimers and even monomers of (+)-catechin or (–)-epicatechin as above mentioned, all of which are reported as absorbed in the small intestine (33). These compounds are subsequently glucuronidated, methylated, and sulfated in the liver, reaching maximal concentrations between 19 and 359 nmol/L in the plasma of humans (33).

**Table 3:** Chemical composition of proanthocyanidins (mg/100 g of SP)^a^ determined in the sunflower seed hull powder (SP).

	**DPn**	**Total proanthocyanidin (mg/100 g of SP)**	**EPe (%)**	**CTt (%)**	**EPt (%)**
SP	1.25 ± 0.01	168 ± 5	26.8 ± 0.3	35.1 ± 3.4	38 ± 3
Peak number			**1**	**2**	**3**

* *The mean and standard deviation (n = 2) are reported*.

*DPn, mean degree of polymerization; EPe, epicatechin extension units; CTt, catechin terminal units; EPt, epicatechin terminal units*.

By considering the UV wavelength of absorption and the ions obtained and analyzed through HPLC-ESI-MS (Figure 2C; Table 2), the phenolic compounds released after alkaline hydrolysis included mainly monomers of 3-methoxy-4-hydroxycinnamic acid or ferulic acid (34 mg/100 g SP) (peak 6), and lower amounts of trimers (15.4 mg/100 g SP) (peak 8) and, especially, of dimers (3.5 mg/100 g SP) (peak 7). This indicates that ferulic acid was the phenolic component esterified with the pectins and/or hemicelluloses of SP (34). The total content of esterified ferulic acid was 52.9 mg/100 g SP, as calculated from the composition summarized in Table 2. Regarding this, it has been reported that the *O*-3 of the arabinose residues of hemicelluloses such as xylans, as well as both the arabinose and galactose residues of the rhamnogalacturonan I of pectins, can be esterified by the ferulic acid residue, contributing to the insolubilization of these polysaccharides. Lignin can be also covalently crosslinked by ferulic acid (35).

### Sunflower Seed Hull Film Composites

#### General Characteristics

**Figure 3:**
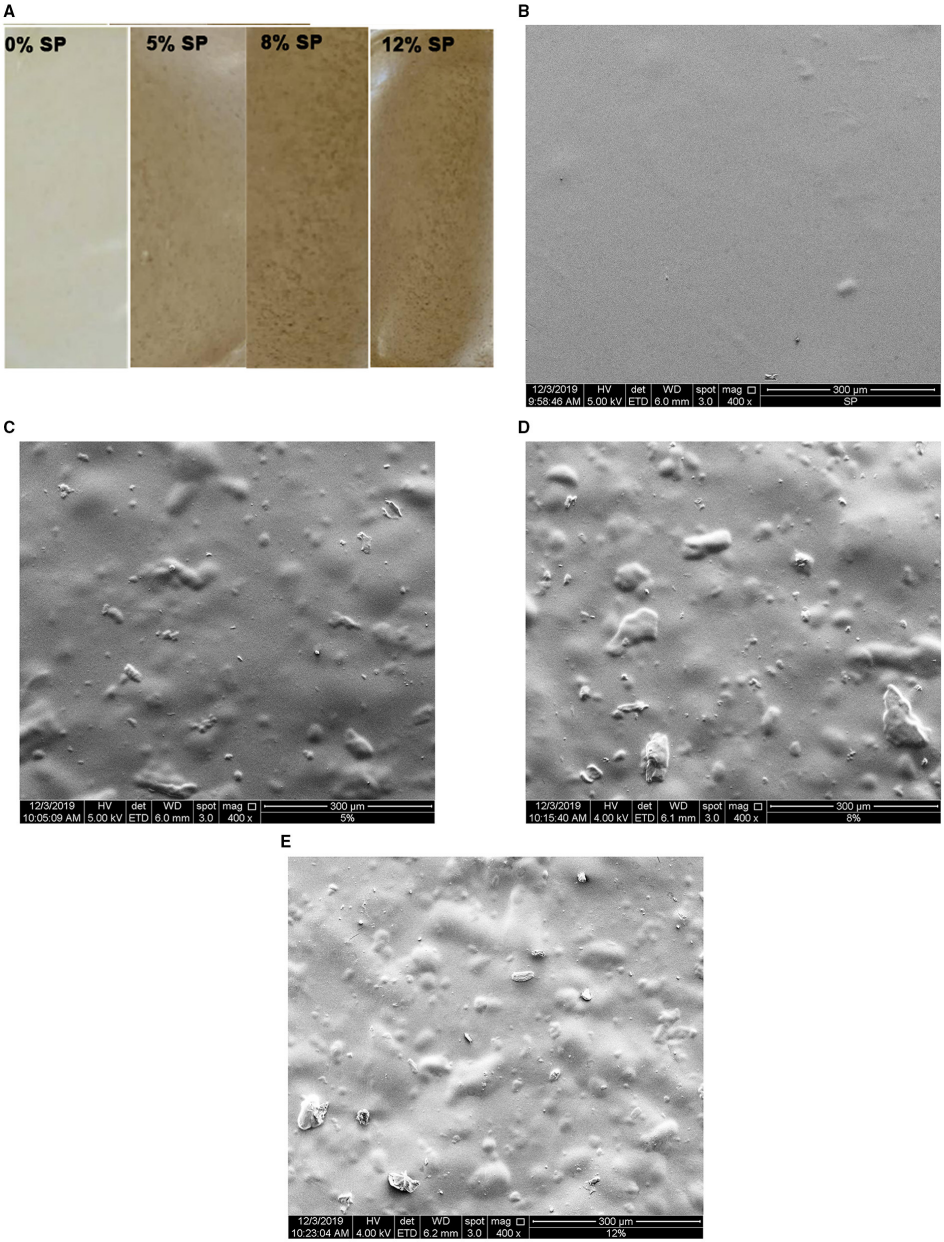
Low methoxyl pectin films loaded with sunflower seed hull powder (SP) of 53 μm of average mesh particle size: 0, 5, 8, and 12% SP **(A)**. The surface morphologies of films loaded with 0% **(B)**, 5% **(C)**, 8% **(D)**, and 12% **(E)** of 53-μm SP at 400X of magnification. The white bar scale at the bottom of each image corresponds to 300 μm.

As described above, film composites were developed with the fraction of SP of 53 μm of average mesh particle size (Figure 1A) as the filler, in proportions of 0, 5, 8, or 12 g/100 g low methoxyl pectin. The film (0% SP) and composites (5, 8, and 12% SP) obtained after casting were flexible and also resistant to handling, showing uniformly dispersed particles in the composites (Figure 3A). In relation to the Hunter Lab color parameters, lightness (*L*) decreased from 81 to 73% with the presence of the SP particles, and even down to 65% as the SP concentration in the composites increased (Table 4). The 0% SP film was slightly yellow (*a* = −1.27; *b* = +14.63) and transparent (Figure 3A), and these color parameters changed slightly with the increasing concentration of SP (Table 4).

**Table 4:** Physical properties*^a^^,^^b^* including the Hunter Lab color parameters and antioxidant capacity^a^^−^^c^ determined on 57.7% equilibrated low methoxyl pectin films made without (0%) or with increasing concentrations of 53-μm sunflower seed hull powder (SP).

	**Composite films (SP % w/w)**				**SP equilibrated at 57.7% RH**
	**0**	**5**	**8**	**12**	
Thickness (mm)	0.113 ± 0.004^A^	0.121 ± 0.006^AB^	0.128 ± 0.011^BC^	0.139 ± 0.006^C^	–
*L* (%)	81 ± 5^A^	73 ± 2^B^	68 ± 2^C^	65 ± 1^D^	–
*a*	−1.3 ± 0.7^A^	+0.16 ± 0.19^B^	+0.89 ± 0.27^C^	+1.42 ± 0.18^D^	–
*b*	+14.6 ± 0.8^A^	+15.9 ± 0.3^B^	+16.2 ± 0.3^BC^	+16.2 ± 0.2^C^	–
Water content (g/100 g dm)	46.5 ± 0.2^A^	44.1 ± 0.3^B^	43.3 ± 0.2^B^	42.0 ± 0.2^C^	–
Glass transition temperature, *T*_g_ (°C)	−80.74	−94.64 27.81	−99.83 −78.90 26.59	−99.70 −79.74 67.39	−87.96 14.23 59.39
Change in specific heat at the glass transition [J.g^−1^ (dm).K^−1^]	1.359	1.233 2.777	0.788 1.326 2.363	0.843 1.401 2.574	2.381 0.075 0.05119
Contact angle (°) measured at 5 s	22 ± 3^AB^	29 ± 9^A, C^	24 ± 4^A, C^	26 ± 3^CB^	–
Contact angle (°) measured at 60 s	16 ± 4^A^	24 ± 9^A^	17 ± 4^A^	22 ± 3^A^	–
WVP × 10^10^ (g·m^−1^·s^−1^·Pa^−1^) (25°C; 0%/70% RH)	13.7 ± 0.1^A^	9.2 ± 0.3^B^	9.8 ± 0.1^B^	9.7 ± 0.1^B^	–
DPPH^3^ (mg AA/100 g film)	ND^A^	12.9 ± 0.6^B^	27 ± 6^C^	32 ± 2^AB^	–
FRAP^3^ (mg AA/100 g film)	23 ± 3^A^	35 ± 4^B^	38 ± 1^BC^	42.6 ± 0.2^C^	–

*The glass transition results obtained from SP are also summarized*.

a *Mean and standard deviations for n = 3 (n ≥ 12 for color parameters) are reported*.

b *The same capital letter as superscript of data in a given row means non-significant differences (p < 0.05)*.

c *Results of the DPPH and FRAP assays are expressed as L-(+)-ascorbic acid (AA), which was used as standard*.

*Dm, dry mass; ND, non-detectable; RH, relative humidity (Equation 1)*.

#### Thickness, Water Content, and T_g_

After equilibration at 57.7% of RH (Equation 1), the film thickness increased for proportions of SP above 5 g/100 g low methoxyl pectin, for the same weight of film-making solution poured into each mold (Table 4). Hence, after the inclusion of the filler, the volume of the film network increased per gram of film-making solution, and thus the thickness increased. The ultrastructure observed through SEM revealed a homogeneous, continuous, smooth, and flat surface in the low methoxyl pectin matrix of the 0% SP film (Figure 3B). A smooth and continuous pectin matrix was also observed in the 5–12% SP composites. This matrix included the SP particles of filler distributed and covered by the pectin matrix, consequently showing a wavy surface (Figures 3C–E). The number of particles per unit area of the image increased with the filler concentration, and particles locally increased the film thickness (Table 4). SEM images show that the aspect ratio (L/D: length/diameter) of the SP particles included in the continuous pectin matrix was highly variable (Figures 3C–E).

The water content determined in the film and composites equilibrated at 57.7% RH (film a_W_ = 0.577; Equation 1) decreased slightly but significantly (*p* < 0.05) from 46.5 g water/100 g dry mass for the 0% SP film down to 44.1 g water/100 g dry mass for the 5% SP film composite, and to 42 g water/100 g dry mass for the 12% SP film composite (Table 4). For the same weight of film-making solution poured into each mold, the presence of SP finally produced an equilibrated film with a water activity of 0.577 and lower adsorption capacity of water (Equation 1). Composites had a lower proportion of the glycerol plasticized hydrophilic pectin network and increasing proportions of the hydrophobic SP, as estimated from the chemical composition of the powder (Table 1) and its hydration properties (Figure 1B). Wahyuningsih et al. (36) developed composite films with water soluble polyvinyl alcohol (PVA) (10% w/v in film forming solution) and pineapple leaf nanocellulose fibers (10–50% w/w in film forming solution) for improving the physical, thermal and barrier properties. The water content of composites maintained at 0% RH decreased from 6.03 to 2.98–4.58% with the increasing proportion of nanocellulose fibers when glycerol was absent. In the presence of glycerol, the water content decreased to 5.62% for composites containing 30–50% of nanocellulose fiber. Simultaneously, the thickness decreased from 0.11 mm for the film made with PVA to 0.03–0.02 mm for all films containing nanocellulose fibers, both without and with glycerol. Probably, as the nanocellulose fibers can interact closely through hydrogen bonds with the hydroxyl groups of the PVA macromolecules, it led to a continuous matrix constituted by both components, and hence with a lower volume occupied. On the other hand, Zolfi et al. (37) developed nanocomposite films composed of kefiran polysaccharide-whey protein isolate (50:50 v/v) in their matrix and montmorillonite (1, 3, and 5% w/w on kefiran–whey protein isolate) as filler, using glycerol as a plasticizer. This hydrated alumina silicate thin layered clay has a high surface area and aspect ratio (L/D). The water content of films decreased deeply with the presence of 1% of montmorillonite, and furthermore with increasing proportions of clay. This result was ascribed by the authors to the strong hydrogen bonds formed between montmorillonite and the macromolecular chains of kefiran-whey protein isolate, which decreased the diffusion of water molecules into the matrix. On the other hand, the film thickness increased slightly but significantly from 0.074 to 0.076 mm with the addition of clay, and also with the clay proportion (0.076–0.091 mm). Jannatyha et al. (38) casted carboxymethyl cellulose films with either nanochitosan or nanocellulose (0.1, 0.5, and 1% w/w). In both cases, the water content of carboxymethyl cellulose film equilibrated at 53% RH decreased from 22 to 14% as the concentration of nanofillers increased, especially with nanochitosan or with 0.5–0.5% nanochitosan-nanocellulose. The film thickness increased significantly from 0.013 mm for carboxymethyl cellulose film up to 0.017 mm with 1% of nanochitosan or nanocellulose, which was ascribed to the higher solid volume. Abdollahi et al. (39) casted glycerol plasticized alginate-based nanocomposites containing cellulose nanowhiskers (1, 3, 5, and 10% w/w on sodium alginate). The water content decreased from 4.70 to 3.08–3.25% (on total base) as the nanocellulose particles increased to 3–10%. The film thickness was not reported.

As reported in Table 4, the glass transition temperature (*T*_g_) determined in the 0% SP film equilibrated at 57.7% RH (Equation 1) was −80.74°C, while, for composites, this low value of *T*_g_ decreased to −94.64, −99.83, and −99.70°C for 5, 8, and 12% SP, respectively. These low values of *T*_g_ can be ascribed to the glycerol plasticization (95.2 g glycerol/100 g low methoxyl pectin), and secondarily to the water content due to film equilibration at 57.7% RH (Equation 1; Table 4). The change in the specific heat at each glass transition was of the same order [1.233–1.359 J.g^−1^ (dm).K^−1^] (Table 4). In the presence of increasing proportions of SP, even when the water content decreased, the *T*_g_ values decreased (Table 4), probably due to an enrichment of the pectin matrix in glycerol. Even though the water content did not change, Bernhardt et al. (40) also observed a decrease in the *T*_g_ values from −64.89 to −71.83°C as the proportion of the 53-μm water insoluble fiber particles obtained from corn husks increased from 1 to 8%, in the 57.7% RH equilibrated composites based on low methoxyl pectin. In other works, temperatures scanned through DSC were from −50 to 250°C (37), from 25 to 300°C (38, 41), and from 30 to 480°C (36), and then reported *T*_g_ values where the effect of the plasticizer at very low temperatures was not demonstrated. In this sense, Panova et al. (41) scanned at 3°C/min from 25°C to determine the glass transition temperature of PVA-graphene oxide (2% or 4% w/w) nanocomposites through thermomechanical analysis. These authors (41) observed a decrease of *T*_g_ from 94°C for PVA films to 75 and 42°C for PVA-graphene oxide (2% or 4% w/w) nanocomposites made with increasing contents of glycerol. In alginate films loaded with L-(+)-ascorbic acid, De'Nobili et al. (42) determined that the *T*_g_ decreased from −40.06 to −63.37°C and from −40.06 to −66.83°C as both the glycerol proportion and RH (33.3, 57.7, and 75.2%) of equilibration increased.

In our composites containing the highest SP concentrations, 8 and 12%, other low *T*_g_ values were, respectively, found, −78.90 and −79.74°C (Table 4). All these low *T*_g_ values also corresponded to plasticized regions of the film (0% SP) and composite networks, which are amorphous-rubber regions at room temperature. The composites also showed a *T*_g_ value above the room temperature (25°C), which increased from 27.81 or 26.59°C for the 5 and 8% SP networks and up to 67.39°C for the 12% SP network (Table 4). These values reveal the existence of glassy regions in the composites, which can be ascribed to the SP polymeric filler particles distributed in the low methoxyl pectin matrix (Figures 3C–E).

When the 53-μm SP powder was separately equilibrated at 57.7% RH and then submitted to the DSC scan at the same conditions of films (10°C/min), a low *T*_g_ of −87.96°C was observed (2.381 J/g of c_P_) (Table 4). This value was higher than the lowest *T*_g_ (−94.64 and −99.70°C) observed in composite films, but lower than the next *T*_g_ value (−78.90°C; −79.74°C) also observed in composite films (Table 4). This *T*_g_ of SP powder can be ascribed to the most hydrophilic polysaccharides (pectins, hemicelluloses) plasticized by the adsorbed water. The other two values of *T*_g_, 14.23 and 59.39°C, determined in the 53-μm SP powder (Table 4) also corresponded, respectively, to amorphous-rubber regions at room temperature due to the presence of polysaccharides plasticized by water (pectins, hemicelluloses, and amorphous cellulose), and to the existence of amorphous-glassy regions of ordered polysaccharides, such as cellulose microfibrils of the SP cell walls and lignin (43).

Polymer blends are mixtures of multiple types of polymers, such as the mixtures of cellulose, hemicelluloses, and lignin in the cell wall, and low methoxyl pectins in films. Their *T*_g_ values depend on the *T*_g_ of the individual polymers, and their miscibility with each other (43). In the present study, no first-phase transitions were observed in the thermograms, indicating that no crystalline or highly ordered polysaccharides (e.g., crystalline cellulose) were detectable in 57.7% RH equilibrated SP powder (43). The *T*_g_ values found in 53-μm SP powder were different from those revealed in the SP composites (27.81, 26.59, and 67.39°C) (Table 4). These different results can be expectable since the treatment of the isolated 53-μm SP powder equilibrated at 57.7% RH (Table 4) was not the same as that received by the 53-μm SP powder included as filler in the low methoxyl pectin network of composites. In the latter case, SP particles were hydrated for 24 h before being added to each film-making solution of low methoxyl pectin, which was then heated up to 90°C, after which calcium, glycerol, and potassium sorbate were added. Each of these whole hydrated systems was then cooled at 8°C for gelling into plates and then dried under forced convection at 60°C for 2.5 h (see Film Preparation section). Hence, the final polymer state of the filler particles of SP included in the low methoxyl pectin matrix (Figures 3C–E) was necessarily different from that evaluated in the isolated 53-μm SP powder, even after equilibration at 57.7% RH (Table 4). Moreover, filler particles at an ideal concentration can be able to form a filler-interconnected network in composite films, as previously observed in Bernhardt et al. (40) for 53-μm corn husk fiber at 5% concentration.

#### Contact Angle, WVP, and Mechanical Properties

The values of the contact angle at the film surface were well below 90°, and with a non-significant trend to increase with the SP concentration, especially for the 5% SP film (Table 4). Therefore, water wets the film (0% SP) and composites. Except for the 5% SP film, the contact angle decreased after 60 s of water drop permanence (Table 4). These values were similar to those obtained by Bernhardt et al. (40) for films loaded with 53-μm corn husk fiber at proportions of 1, 3, and 8% w/w, except for the 5% fiber composite whose contact angle was 44° and decreased to 36° after 60 s of water drop permanence. According to the mechanical results, at this filler concentration, the formation of a 53-μm corn husk fiber interconnected network was proposed (40). Abdul Khalil et al. (44) casted films from a 2% w/v dry seaweed film forming solution containing either 0, 1, 5, 10, 20, or 30% w/w (on dry seaweed) of dried oil palm shell (2.0–2.8 μm average particle size). The contact angle of the seaweed film (62.3°) decreased with the filler content increase, being 47.25° the lowest value reached, which was for the 20% filler composite. This result was attributed to the hydrophilic character of the oil palm shell microparticles.

The WVP, evaluated at 25°C under convection in a 0–70% RH gradient according to Gennadios et al. (24), decreased slightly but significantly (*p* < 0.05) for composite films (9.8 × 10^−10^ g·m^−1^·s^−1^·Pa^−1^) with respect to the film made only with low methoxyl pectin (Table 4). The 53-μm SP particles may constitute filler obstacles for water vapor diffusion across the composite networks, and also probably decreased the SC of the hydrophilic low methoxyl pectin matrix. The effect of water plasticization (as well as that of glycerol in the case of films) is the primary reason for moisture effects on diffusion. In polymer assemblies with hydrogen bonding, water can act as a plasticizer, being the primary reason for moisture effects on diffusion in cell walls (43). Bernhardt et al. (40) also observed a significant decrease in the WVP (25°C; 0–70% RH gradient) from 11 × 10^−10^ to 8.0 × 10^−10^ g·m^−1^·s^−1^·Pa^−1^ when the low methoxyl pectin film was loaded with 3–8% of 53-μm corn husk fiber, and obtained WVP values similar to those determined in the present work (Table 4). Jannatyha et al. (38) observed that the WVP (25°C; 0–52.8% RH gradient) of carboxymethyl cellulose films (0.78 × 10^−10^ g·m^−1^·s^−1^·Pa^−1^) also decreased with the increase of either nanochitosan (0.45–0.17 × 10^−10^ g·m^−1^·s^−1^·Pa^−1^) or nanocellulose (0.37–0.28 × 10^−10^ g·m^−1^·s^−1^·Pa^−1^) concentration loaded (0.1, 0.5, and 1% w/w) or with 0.5–0.5% nanochitosan-nanocellulose (0.11 × 10^−10^ g·m^−1^·s^−1^·Pa^−1^). These WVP values were lower than those determined in our work (Table 4), in part due to the lower RH gradient used by Jannatyha et al. (38).

**Figure 4:**
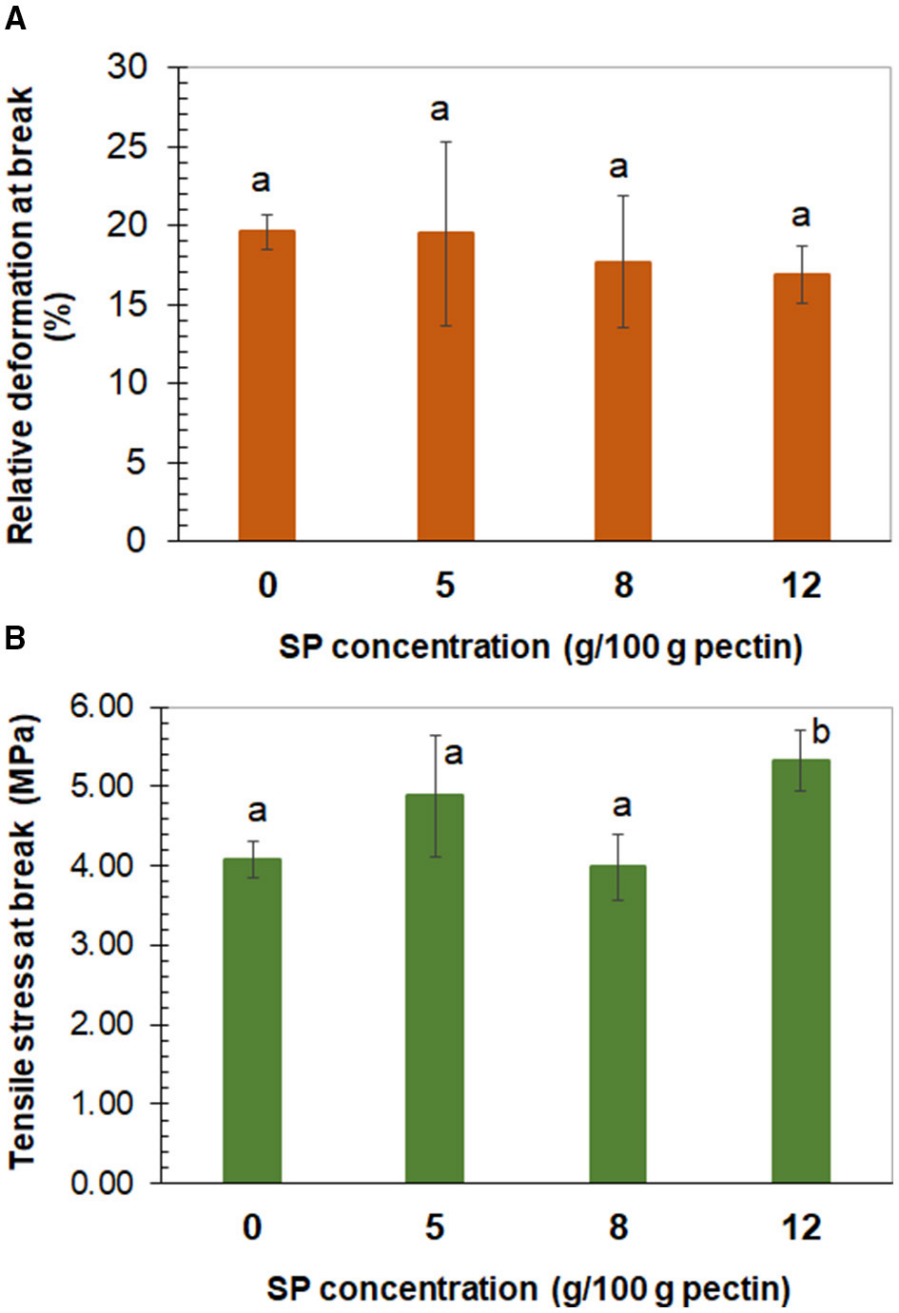
Tensile relative deformation **(A)** and tensile stress **(B)** at break determined in low methoxyl pectin films, plotted against the concentration of sunflower seed hull powder (SP) of 53 μm of average mesh particle size. **(A)** The same lowercase letter over the bars means non-significant differences (*p* > 0.05) among bars. **(B)** The same lowercase letter over bars means non-significant differences (*p* > 0.05) among these bars. Error bars correspond to the standard deviation (*n* ≥ 10).

Through the uniaxial tensile assay, the film (0% SP) and composites equilibrated at 57.7% RH showed the same relative deformation or strain at fracture (16–19%; Figure 4A). At the same time, significantly (*p* < 0.05) higher tensile stress at break was observed for the 12% SP film composite (5.3 MPa), whereas non-significantly different tensile stress at break was observed for the 5% SP film composite (4.9 MPa) with respect to the other systems (4.0 MPa) (Figure 4B). The 12% concentration of 53-μm SP particles in the low methoxyl pectin matrix seemed to be the lowest concentration where a net increase in tensile stress was observed. Higher stress at break reflects higher strength of interaction due to adhesion between the filler particles and the film matrix. In the case of no adhesion, the interfacial layer cannot transfer stress (45). Interfacial interaction between filler and matrix is an important factor affecting the mechanical properties of the composites. It depends on the area of the interphase and on the strength of the interaction. The characteristics of the filler surface affect the interfacial interaction (45). Bernhardt et al. (40) also observed non-significant difference in the strain at fracture determined (≈9%) for low methoxyl pectin film and 53-μm corn husk fiber (1–8%) composites. Simultaneously, the significantly (*p* < 0.05) highest tensile stress was determined in the film loaded with 5% of fiber (140 MPa) and the lowest one (≈58 MPa) in the 1 and 8% corn husk fiber composites. On the other hand, Jannatyha et al. (38) observed that the strain at break of carboxymethyl cellulose films (201%) decreased with the increase of either nanochitosan (140–29%) or nanocellulose (101–70%) concentration loaded (0.1, 0.5, and 1% w/w) or with 0.5–0.5% nanochitosan-nanocellulose (4.96%). On the contrary, the corresponding tensile stress at break of carboxymethyl cellulose films (6.10 MPa) increased with the filler concentration, either of nanochitosan (8.41–18 MPa) or nanocellulose (7.23–12.3 MPa) or with 0.5–0.5% nanochitosan-nanocellulose (9.95 MPa). In the case of nanochitosan, the highest stress at break (18 MPa) corresponded to 0.5% concentration and decreased to 11.3 MPa for 1% of nanochitosan due to self-agglomeration. According to the authors, the electrostatic interaction between the carboxyl groups of carboxymethyl cellulose and amino groups of chitosan nanoparticles justified the adhesion between the matrix and the filler, and higher tensile stress.

#### Antioxidant Capacity

The antioxidant capacity of the film and composites was evaluated through their free radical scavenging capacity (DPPH assay) and ferric reducing capacity (FRAP assay) and expressed as AA (Table 4). The DPPH activity was absent in the 0% SP film and increased with the SP concentration, from 12.9 to 32 mg of AA per 100 g of the film (Table 4). Instead, the components of the 0% SP film showed ferric reducing capacity (FRAP assay), which increased significantly (*p* < 0.05) from 35 to 42.6 mg of AA per 100 g of film as the SP concentration in composites increased (Table 4). Polysaccharides such as pectins retard lipid oxidation in oil-in-water emulsions due to metal ion chelation and hydrogen donation (46, 47). The latter can explain the FRAP demonstrated by the low methoxyl pectin film with 0% SP (Table 4). The composite films developed with the SP filler can then be proposed as active antioxidant interfaces, which can be ascribed to the phenolic compounds contained in the sunflower hulls (Tables 2, 3). This result enables us the study of these composites as antioxidant film barriers in foods vulnerable to oxidation such as dairy products (13), plant-based cheese analogs (10), poultry meat (48), and beef patties. The appearance of films changed with the increase of 53-μm SP concentration (Figure 3A). These films are edible as inferred from the very low value of chronic toxicity (1 g/mL) of the sunflower hull (6). Edible films based on polysaccharides are in general no waterproof materials and, hence, can not be used as unique packaging films. Also, edible films based on polysaccharides are in general not thermo-sealable (9). Therefore, the 12% SP film could be suggested as a food slice separator for antioxidant preservation, which will be investigated in a future work.

## Conclusions

Sunflower hulls remaining after oil extraction, reduced to a powder (SP), constituted mainly by 210 μm (51.4%) and 420 μm (27.6%) average mesh sizes particles, contained ≈30% of cellulose, 26.4% of lignin, 38.5% of neutral sugars, mostly ascribed to hemicelluloses, and only 1.3% of proteins. The important lignin content and low pectin content (4.0% of uronic acids) were associated with the low hydrophilic behavior of the powder and, hence, the poor hydration capacity. Phenolic compounds were mostly proanthocyanidins (168 mg/100 g SP) of low DPn (1.25), with lower amounts of extractable (31.4 mg/100 g SP) phenolics (*O*-*p*-caffeoylquinic acid, 23.7 mg/100 g SP) and esterified ferulic acid (52.9 mg/100 g SP) monomers and trimers. Proanthocyanidins, as well as extractable phenolics of intracellular origin, can be responsible for the relevant DPPH radical scavenging capacity (95 mg AA/100 g) and FRAP (152 mg AA/100 g) of SP. These compounds imparted antioxidant capacity in a concentration-dependent manner to the composite films loaded with 53-μm particle size SP (0, 5, 8, and 12% SP) based on calcium-crosslinked low methoxyl pectin. The WVP decreased, while the normal stress at film fracture increased significantly for 12% SP composites which meant higher strength of interaction due to adhesion between the filler particles and the pectin matrix. Our results allow concluding that sunflower hulls remaining after oil extraction constitute a valuable source of biopolymers and co-extracted phenolics for the development of active materials such as composite films for antioxidant preservation at food interfaces, which will be further investigated.

## Data Availability Statement

The original contributions presented in the study are included in the article/supplementary material, further inquiries can be directed to the corresponding author.

## Author Contributions

MD: experimental work, data collection, data analysis, methodology, and writing of the original draft. DB: collaboration in the experimental work. MB: direction of experimental chemical analysis through HPLC, data analysis, and writing revision. AR: direction of the work, supervision, data analyses, writing and editing, project administration, and funding acquisition. All authors contributed to the article and approved the submitted version.

## Funding

This study was financed by grants from the University of Buenos Aires (2018–2021, 20020170100229BA) and Agencia Nacional de Promoción Científica y Tecnológica (ANPCyT) (PICT 2015-2109; PICT 2017-1146), Argentina.

## Conflict of Interest

The authors declare that the research was conducted in the absence of any commercial or financial relationships that could be construed as a potential conflict of interest.

## Publisher's Note

All claims expressed in this article are solely those of the authors and do not necessarily represent those of their affiliated organizations, or those of the publisher, the editors and the reviewers. Any product that may be evaluated in this article, or claim that may be made by its manufacturer, is not guaranteed or endorsed by the publisher.
